# Patient attributes warranting consideration in clinical practice guidelines, health workforce planning and policy

**DOI:** 10.1186/1472-6963-11-221

**Published:** 2011-09-19

**Authors:** Matthew J Leach, Leonie Segal

**Affiliations:** 1 Health Economics and Social Policy Group, University of South Australia, Adelaide, South Australia

## Abstract

**Background:**

In order for clinical practice guidelines (CPGs) to meet their broad objective of enhancing the quality of care and supporting improved patient outcomes, they must address the needs of diverse patient populations. We set out to explore the patient attributes that are likely to demand a unique approach to the management of chronic disease, and which are crucial if evidence or services planning is to reflect clinic populations. These were incorporated into a new conceptual framework; using diabetes mellitus as an exemplar.

**Methods:**

The patient attributes that informed the framework were identified from CPGs, the diabetes literature, an expert academic panel, and two cross-disciplinary panels; and agreed upon using a modified nominal group technique.

**Results:**

Full consensus was reached on twenty-four attributes. These factors fell into one of three themes: (1) type/stage of disease, (2) morbid events, and (3) factors impacting on capacity to self-care. These three themes were incorporated in a convenient way in the workforce evidence-based (WEB) model.

**Conclusions:**

While biomedical factors are frequently recognised in published clinical practice guidelines, little attention is given to attributes influencing a person's capacity to self-care. Paying explicit attention to predictable threats to effective self-care in clinical practice guidelines, by drawing on the WEB model, may assist in refinements that would address observed disparities in health outcomes across socio-economic groups. The WEB model also provides a framework to inform clinical training, and health services and workforce planning and research; including the assessment of healthcare needs, and the allocation of healthcare resources.

## Background

The development of clinical practice guidelines (CPGs) has transformed significantly over the past decade. Guidelines are increasingly less dependent on consensus of expert opinion, and more reliant on published evidence from randomised controlled trials [1]. The intention of CPGs has also widened. Not only are guidelines designed to inform clinicians about the appropriate management of specific health conditions, they are also intended to 'improve the quality of health care...reduce the use of unnecessary, ineffective or harmful interventions, and...facilitate the treatment of patients with maximum chance of benefit, with minimum risk of harm, and at an acceptable cost' [2].

Whilst the growing emphasis on evidence-based practice is welcome, it is not without its challenges. Evidence hierarchy's privilege randomised controlled trials to maximise internal validity, but this can be at the cost of external validity. This occurs where trial populations are narrowly defined by inclusion and exclusion criteria, so that they do not well represent the patients seen in clinical practice. Participants recruited into clinical trials often differ from the patient population in terms of education level, social class, age, race and disease severity; this makes translating evidence-based CPGs into clinical practice and policy problematic [3]. That this is an issue is indicated by clinician concerns with the usefulness of CPGs, and the lack of breadth and depth of CPGs.

A systematic review of thirty surveys investigating clinician attitudes to CPGs supports the abovementioned position, with many physicians concerned that clinical guidelines are not applicable to individual patients [4]. According to a review of 28 CPGs of nine chronic diseases typically managed in primary care [5], little consideration is given to the patient with complex co-morbid illness, or to pertinent psychosocial elements such as culture, education level, socioeconomic status, patient preference and burden of treatment [5].

Given that these biological, psychological and social factors may impact on the best approach to [6], and effectiveness of patient care, neglecting these elements in clinical practice could result in poorer patient outcomes [5]. Furthermore, failure to understand and describe the patient population, in relation to the biopsychosocial attributes that are critical to the preferred approach to care, could in the context of health services and health workforce planning, lead to (1) the under-provision of necessary clinical services, and (2) primary care teams with insufficient skill-mix to adequately address patient needs [1].

This paper considers which patient attributes are likely to demand a unique approach to management; in terms of objectives of care, membership of the care team, and pattern of clinical appointments (e.g. frequency and duration of consultations). We also describe a conceptual framework devised to assist clinicians, educators, researchers, authors of CPGs, health services and health workforce planners to assess and handle the diverse patient populations seen in clinical practice.

Diabetes mellitus was chosen as a case exemplar to explore this issue; as a chronic and debilitating disease of high and increasing prevalence across the globe, for which adoption of best practice care has been demonstrated to improve outcomes, and for which there is considerable diversity in patient population across a range of factors that might be expected to impinge on appropriate management.

## Methods

The project set out to develop a set of agreed population attributes that are important in clinical practice for chronic diseases (using diabetes as an exemplar), and would ideally be incorporated in some way into clinical practice guidelines and health services policy formulation. A four-stage approach was taken to ascertain which attributes of people with diabetes may necessitate a significantly different approach to management.

### Stage 1: Review of clinical practice guidelines

A systematic search of MEDLINE, EMBASE, CINAHL, Scopus and the grey literature for all CPGs relating to the management of type 1, type 2 and/or gestational diabetes in the primary health care setting was conducted. The search, which was limited to guidelines published in the English language between 2003 and 2009 (to ensure guidelines were relevant), served to identify a list of patient attributes for which distinct management objectives were described. The scope and depth of the guidelines in covering specific sub-populations was assessed against Appraisal of Guidelines Research and Evaluation (AGREE) collaboration criteria [7]. The methodology and outcomes of stage 1 are described in detail elsewhere [1].

### Stage 2: Review of the clinical literature

The clinical literature on the management of diabetes was systematically searched for any additional patient attributes potentially relevant to diabetes management (searching until no new characteristics emerged). The search was conducted across three bibliographic databases (including MEDLINE, EMBASE and CINAHL), using the following search terms:

1. Diabetes mellitus OR type 1 diabetes mellitus OR type 2 diabetes mellitus OR gestational diabetes

2. Self-care OR self-efficacy OR patient compliance OR health status OR health behaviour OR treatment outcome

3. 1 AND 2.

The search was limited to papers published in the English language after the year 1990 (to capture only the most recent literature), and for which an abstract was available. Publications investigating the prevalence, incidence, prevention or screening of diabetes and diabetes complications, or the efficacy of pharmaceutical agents, were excluded.

### Stage 3: Input from expert academics

Discussions were held with an expert academic panel to identify any additional patient attributes warranting substantial consideration in the management of diabetes mellitus in the primary care setting. The panel was selected by invitation, and comprised a diabetologist, diabetes educator, community nurse, dietician, podiatrist, cardiologist, occupational therapist and a public health physician; all were located in metropolitan Adelaide, and all had clinical expertise in managing diabetes.

### Stage 4: Cross-disciplinary consensus

A second and third cross-disciplinary panel of clinicians who worked directly with patients with diabetes in Metropolitan Adelaide (South Australia) or the regional centre of Whyalla (South Australia), respectively, were conducted. Two geographically diverse panels were formed as it was suspected that patient attributes may vary across metropolitan and regional settings. The Metropolitan panel comprised a diabetologist, diabetes educator, practice nurse, dietician, diabetes counsellor, social worker, pharmacist, community nurse, podiatrist, general practitioner, exercise physiologist and occupational therapist. The regional panel included a diabetes educator, dietician, social worker and pharmacist. The aim was to seek confirmation or adjustment to the patient attributes identified through the prior processes, using a modified nominal group technique [8]. Effectively, clinicians commented on the subpopulations in isolation and without influence from other panel members. Clinicians then conveyed their suggestions during the panel meetings, and discussed the ideas presented. Findings were iterated to a point of consensus; determined by way of voting (an attribute was accepted if more than seventy-five percent of the panel supported the attribute, and there were no strong rejections to its inclusion). Patient attributes were then grouped into logical and meaningful themes using inductive reasoning, and later developed into a conceptual framework, which was workshopped with the cross-disciplinary panels. While this approach was developed within a particular geographical setting, the outcomes almost certainly have broader application, interstate and internationally.

## Results

**Figure 1:**
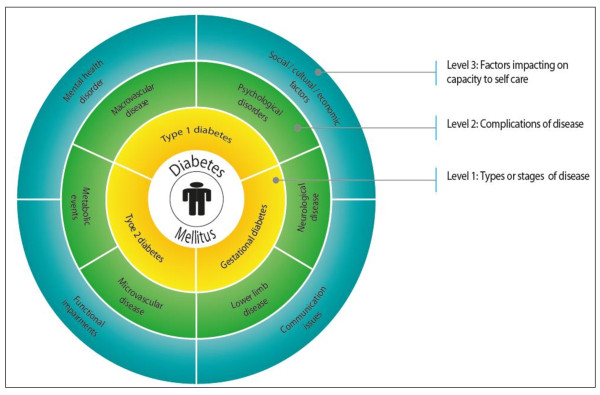
Workforce evidence-based (WEB) model* for diabetes mellitus. *The workforce evidence-based (WEB) model assists clinicians, researchers, educators, and health services and workforce planners to recognise, and effectively manage, the complex needs of individual patients with chronic disease. The model takes into account three key elements that necessitate a significantly different approach to the management of chronic disease, including: (i) types and/or stages of the disease, (ii) complications and/or morbid events associated with the disease, and (iii) factors impacting on the patient's capacity to self-care. Essentially, a person with chronic disease may experience one or more of these factors, at any period of time, throughout the life of the disease - accounting for literally millions of individual patient types; each with distinctly different care and service requirements.

The expert panels and research team identified thirty-five attributes that may demand a unique approach to the management of diabetes in the primary care setting. The search of the bibliographic databases for pertinent attributes and diabetes CPGs found 2,140 and 4,278 citations, respectively; from which fourteen and seventeen attributes were extracted, respectively. Excluding duplicates, a total of thirty-nine distinct attributes were identified. Full consensus was reached on the inclusion of twenty-four of these attributes. These attributes were grouped into three distinct themes, which also form the basis of the conceptual framework for chronic disease management; hereon referred to as the Workforce Evidence-Based planning (WEB) model (see Figure 1). The three identified groupings were:

i. Type or stage of disease *(Level 1, or inner circle of the WEB model)*,

ii. Complications of disease *(Level 2, or second circle of the WEB model)*, and

iii. Factors impacting on capacity to self-care *(Level 3, or outer circle of the WEB model)*.

Of the fifteen elements that were rejected, two were considered outside the scope of the project, and twelve were captured within other elements. The remaining factor, age, was the issue of some debate. It was decided that whilst age is likely to influence an individual's ability to self-manage diabetes, the needs of younger and older persons were better addressed through specific threats to self-care capacity, as listed in the outer band of the WEB model (Figure 1); such as social issues (i.e. peer pressure or living alone), functional impairment (i.e. physical ability) or communication issues (i.e. health literacy or cognitive ability).

## Discussion

A recent critical appraisal of 27 CPGs relating to the management of diabetes in the primary care setting [1], found that biomedical factors (i.e. categories i and ii) are frequently recognised in published CPGs (with the exception of sexual dysfunction). Given their wide acceptance, they are not further explored in this paper. Our main contribution in relation to the biomedical attributes is their incorporation into the WEB model, and the additional tractability this offers to the effective consideration of biomedical together with other patient attributes in clinical practice, and health workforce and health services planning.

The factors grouped under threats to self-care capacity (Level 3 of the WEB model [Figure 1]) are as a rule less frequently recognised in clinical guidelines; or less fully explored. The reason for this is unclear, given the weight of evidence identifying the crucial role of effective patient self-care in maintaining good glycaemic control, control of other risk factors, and reducing the rate of complications (as detailed below). A brief discussion of the key attributes identified as potential threats to self-care capacity, about which specific clinical or other service strategies might be needed to enhance care outcomes (and for which adequate recognition in clinical practice guidelines is warranted), is provided below.

### Attributes identified as constituting potential threats to self-care

#### Cognitive ability

Diabetes mellitus is associated with poorer cognitive function [9,10]. These changes are likely to affect a person's capacity to self-manage diabetes; although, the correlation between cognitive ability and glycaemic control has been poorly investigated. Regardless of whether reduced cognitive ability is a complication of diabetes, persons with diabetes and poor cognitive function, particularly those living alone, are likely to require more frequent surveillance, repetitive education and/or exposure to innovative reminder strategies; thus, it seems desirable to identify a specific management approach for this group.

#### Language proficiency

People with a significant language barrier may be at risk of poor care outcomes due to difficulties with communication. To illustrate, a survey of 1,262 patients with type 2 diabetes from the Fremantle diabetes study found residents non-fluent in the English language were significantly less likely to attend diabetes education programs and more likely to demonstrate poorer diabetes knowledge scores than those fluent in English [11]. Findings from a systematic review of 36 published studies suggest that access to professional health interpreters or bilingual providers improves communication, quality of care and clinical outcomes in patients with limited English proficiency; including those with diabetes [12]. These findings indicate that language demands greater consideration in the overall management of diabetes and the introduction of specific strategies.

#### Indigenous, ethnic, racial and cultural background

Race, ethnicity and cultural background may influence, to varying degrees, a person's, lifestyle choices, access to care, and understanding of health and illness; all of which impact on diabetes management. For example, an outpatient survey of 500 diabetes patients in the UK; including 232 Caucasians born in the UK and 268 patients born in the Indian subcontinent, found a significantly lower awareness of diabetes complications, knowledge of diabetes, nutritional content of the diet and perceived importance of controlling diabetes amongst the Indian group [13]. Multivariate analysis demonstrated that these differences were more closely associated with ethnicity than income or education level. Similar findings relating ethnicity to poor diabetes management and knowledge have also been reported in Kashmiri men in England [14]. A systematic review of 51 studies reported worse outcomes of diabetes care, including glycated haemoglobin, and poorer quality of life among patients with diabetes from ethnic minority groups, such as African-Americans [15].

A number of studies examining urban and remote populations of indigenous peoples have shown that indigenous adults with confirmed diabetes have lower rates of diabetes education, insulin treatment, self-monitoring of blood glucose levels, podiatry referrals, and worse mean glycated haemoglobin levels, together with a greater risk of micro- and macro-vascular complications when compared to non-indigenous persons [16,17]. Suggested reasons for these poorer outcomes include poor physician compliance with CPGs, family or cultural needs, affordability and access to culturally appropriate care, education, income, housing and health literacy [16,17]. Considering explicitly the mix of health and welfare workers best able to find solutions to these issues will be important to improving health outcomes in indigenous and ethnic minority groups with diabetes. We note CPGs do recognise the specific needs of indigenous patients, but rarely other ethnic groups.

#### Socioeconomic and social issues

A number of social and economic factors influence the capacity for effective management of diabetes. Several small studies report an association between low socioeconomic status and poor adherence to dietary regimes and physical activity recommendations among adults with type 2 diabetes [18,19]. A large retrospective analysis of four Israeli health maintenance organization databases found that for people with diabetes from low socioeconomic backgrounds, while demonstrating greater adherence to annual diabetes health assessments, exhibited poorer lipid and glycaemic control [20]. Improved diabetes screening among lower socioeconomic groups thus did not translate into better health outcomes.

The capacity of an individual to manage their diabetes can be influenced by a range of social factors, including a person's family and work commitments, the availability of social support, access to secure housing (which impacts on the ability to prepare food and store insulin), income, and access to treatment. In addition is the direct effect of stress (from any of these social and economic factors) on glycaemic control. Studies in adults and adolescents with diabetes report, for instance, that lower social support, single-parent family status and increased family conflict are significantly correlated with reduced treatment compliance and poorer glycaemic control [21,22]. A different approach to care, as well as the provision of additional services, such as counselling or social work, may be fundamental to achieving better outcomes in this subpopulation.

#### Health literacy

Health literacy, or the ability to acquire, comprehend and communicate health information, directly affects an individual's capacity to self-manage diabetes and other health conditions. There is evidence that physician explanations of conditions and processes of care are generally not well tailored to the person with poor health literacy, resulting in a lack of understanding [23]. Not surprisingly, patients with inadequate health literacy demonstrate poorer knowledge of diabetes [24,25]. Nonetheless, the literature is equivocal in terms of the importance of this variable in care outcomes [26,27]. It is also less clear whether this attribute carries particular requirements that differ in important ways from best practice care for the typical patient. In short, the need for separate consideration around health literacy is less clear than for other attributes described in this section. Thus, there is a need to further explore the impact of health literacy on clinical outcomes, as well as the effectiveness of strategies to improve health literacy in clinical practice.

#### Mental wellbeing

Poor mental wellbeing is described as a negative mental state affecting one's ability to live a full and creative life, to effectively self-care, and to flexibly deal with life's inevitable challenges [28]. Many studies report psychological wellbeing to be lower in persons with diabetes compared to healthy controls [29,30], although, this finding is not consistent across all studies [31,32]. There are plausible mechanisms to support causal pathways in either or both direction. There is evidence that reduced psychological wellbeing is associated with higher glycated haemoglobin levels in adolescents with type 2 diabetes [33], with reduced adherence to diet and medication use in adults with type 2 diabetes [34], and is a significant predictor of stroke in elderly persons with either type of diabetes mellitus [35]. It thus seems clear that poor mental wellbeing can lead to poorer health outcomes, including higher rates of diabetes complications [34] and worse glycaemic control [33]. This supports the identification of individuals with poor mental wellbeing and the development of specific management strategies to enhance their outcomes, which may have clinical, workforce and health service implications.

#### Physical ability

The complications of diabetes, including retinopathy, peripheral neuropathy, foot ulceration, stroke and coronary heart disease, are all associated with varying degrees of physical disability [36]. These disabilities may impinge on an individual's capacity to self-manage the disease. Whilst the management of these conditions is well recognised and specified in CPGs, the unique needs of this group in relation to the on-going management of their diabetes also needs to be considered. Physical and intellectual disabilities, such as poor vision and reduced mobility, would be expected to impact on a person's ability to self-manage their diabetes; although, the evidence to date is not consistent [37,38]. These conflicting findings indicate that the association between physical and intellectual disability and diabetes-related outcomes and implications for management warrants further investigation. Regardless, it will be desirable for the clinical team to understand a patient's physical and intellectual capacities and limitations, together with available support, in devising a diabetes management plan.

#### Eating disorder

Eating disorders, such as anorexia nervosa, bulimia nervosa and binge-eating disorder, are characterised by abnormal eating behaviours, and are associated with adverse health outcomes. In person's with diabetes, eating disorders can lead to impaired metabolic control, and increase the risk of diabetic ketoacidosis (DKA), diabetic retinopathy, nephropathy and neuropathy [39-41]. Concurrent diabetes and eating disorder may also elevate the risk of co-morbid anxiety disorder and alcohol abuse [42]; which can further impact on diabetes management. Given that diabetes increases the likelihood of developing an eating disorder (particularly in adults) [42], and dietary management is critical to good glycaemic control, it is paramount that clinicians screen for eating disorders, and work to take action on these issues by involving appropriate specialists in the health care team.

#### Substance abuse

The excessive and maladaptive use of substances, such as alcohol and illicit drugs [43], may have a profound impact on diabetes care and health outcomes. For instance, more than fifty percent of young adults with type 1 diabetes who presented to an Australian teaching hospital with DKA over a ten-month period reported the use of illicit drugs at least 48 hours prior to admission [44]. The relationship between substance abuse and DKA is further supported, albeit weakly, by several case reports and surveys [45,46]. These studies also suggest that the combination of substance abuse and diabetes may contribute to cognitive decline, depression, anxiety, hyperglycaemia and death [45-47]. These adverse effects may be attributed, in part, to the pharmacological activity of these substances; they also could be the result of reduced adherence to treatment [48]. Substance abuse will often reduce a person's capacity to self-care; underlining the need for a unique approach to diabetes management in this group in terms of skill-mix and service requirements.

## Conclusions

The proposition of this paper is that certain patient attributes, particularly those that threaten self-care capacity, are likely to require a different approach and distinct mix of skills or capabilities in the management of diabetes. Failure to recognise and comprehensively address the distinct needs of patients with the identified attributes is likely to lead to the delivery of substandard care and suboptimal clinical outcomes. Thus, it is important that the identified patient attributes that suggest threats to self-care, as well as the commonly identified core health problems (including complications), are incorporated in some way in clinical practice guidelines. The policy implications are considerable, implying for instance expansion to membership of the 'core diabetes primary care team' to include also social work and psychology/mental health nursing, as well as the more traditional diabetes specialists. It also means that in communities where threats to self-care (such as social insults or poor mental health) are more common, higher levels of primary care resourcing will be required to ensure access to best practice care.

The WEB model (Figure 1) described in this paper provides a tractable way of considering the distinct and potentially complex management needs of highly diverse clinic patient populations, not by ignoring important patient attributes, but by adopting a modular approach. Under the modular approach, particular attributes can be selected or omitted according to the unique characteristics of a regional or clinic population (or even individual patient). In this way, the WEB model could be useful for guiding the delivery of individualised patient care (complementing current approaches to care planning/case management); but also for informing guideline development, identifying relevant gaps in research, informing interdisciplinary learning, in addition to the primary role of guiding health services and health workforce planning (i.e. the assessment of healthcare needs, and the allocation of healthcare resources).

We recognise that there are some potential limitations to this research. Firstly, it is possible that selective membership of the expert panels could have unduly influenced the selection of patient attributes. However, we sought to minimise this risk by incorporating the preceding literature reviews (i.e. stages 1 and 2) in the deliberations of the expert panels, as well as using 'rolling panels' with different membership. There also may be concerns that the patient attributes have been defined too broadly and thus, are subject to interpretation. Whilst this is possible, we rather suggest the broad nature of the categories offers flexibility to stakeholders supporting the more meaningful application of the model to diverse populations.

There also will be challenges in applying the WEB model to the wider primary health care workforce, for health services planning across all chronic diseases. It is our recommendation that the three levels and twenty-four patient attributes identified in the WEB model be (a) covered more comprehensively in guidelines to inform clinical practice, and (b) considered in teaching, research, and health services and health workforce planning.

## Declaration of competing interests

The authors declare that they have no competing interests.

## Authors' contributions

ML and LS contributed equally to the writing of the manuscript, from conception to submission. Both authors read and approved the final manuscript.

## Pre-publication history

The pre-publication history for this paper can be accessed here:

http://www.biomedcentral.com/1472-6963/11/221/prepub
